# Thoracic Endometriosis Syndrome (TES) in Martinique, a French West Indies Island

**DOI:** 10.3390/jcm12175578

**Published:** 2023-08-26

**Authors:** Moustapha Agossou, Bruno-Gilbert Sanchez, Paul-Henri Alauzen, Maud Olivier, Elsa Cécilia-Joseph, Ludivine Chevallier, Mehdi Jean-Laurent, Aude Aline-Fardin, Moustapha Dramé, Nicolas Venissac

**Affiliations:** 1 Department of Respiratory Medicine, CHU of Martinique, 97261 Fort-de-France, France; 2 Department of Thoracic and Cardiovascular Surgery, CHU of Martinique, 97261 Fort-de-France, France; 3 Department of Medical Information, CHU of Martinique, 97261 Fort-de-France, France; elsa.cecilia-joseph@chu-martinique.fr; 4 Department of Gynecology and Obstetrics, CHU of Martinique, 97261 Fort-de-France, France; 5 Department of Pathology, CHU of Martinique, 97261 Fort-de-France, France; 6 Department of Clinical Research and Innovation, CHU of Martinique, 97261 Fort-de-France, France; 7 EpiCliV Research Unit, Faculty of Medicine, University of the French West Indies, 97261 Fort-de-France, France; 8 Department of Thoracic Surgery, CHRU of Lille, 59000 Lille, France; nicolas.venissac@chu-lille.fr

**Keywords:** endometriosis, thoracic endometriosis syndrome, catamenial pneumothorax, hemothorax, black people

## Abstract

Introduction: Endometriosis is a female disease that affects 5–10% of women of childbearing age, with predominantly pelvic manifestations. It is currently declared as a public health priority in France. Thoracic endometriosis syndrome (TES) is the most common extra-pelvic manifestation. Objective: The objective of this study was to describe the epidemiological and clinical characteristics, and outcomes of patients with TES in Martinique. Patients and Methods: We performed a descriptive, retrospective study including all patients managed at the University Hospital of Martinique for TES between 1 January 2004 and 31 December 2020. Results: During the study period, we identified 479 cases of pneumothorax, of which 212 were women (44%). Sixty-three patients (30% of all female pneumothorax) were catamenial pneumothorax (CP) including 49 pneumothoraxes alone (78% of catamenial pneumothorax) and 14 hemopneumothorax (22% of catamenial pneumothorax). There were 71 cases of TES, including 49 pneumothoraxes (69%), 14 hemopneumothoraxes (20%) and 8 hemothorax (11%). The annual incidence of TES was 1.1 cases/100,000 inhabitants. The prevalence of TES was 1.2/1000 women aged from 15 to 45 years and the annual incidence of TES for this group was 6.9/100,000. The annual incidence of CP was 1 case/100,000 inhabitants. The average age at diagnosis was 36 ± 6 years. Eight patients (11%) had no prior diagnosis of pelvic endometriosis (PE). The mean age at pelvic endometriosis diagnosis was 29 ± 6 years. The mean time from symptom onset to diagnosis was 24 ± 50 weeks, and 53 ± 123 days from diagnosis to surgery. Thirty-two patients (47%) had prior abdominopelvic surgery. Seventeen patients (24%) presented other extra-pelvic localizations. When it came to management, 69/71 patients (97%) underwent surgery. Diaphragmatic nodules or perforations were found in 68/69 patients (98.5%). Histological confirmation was obtained in 55/65 patients who underwent resection (84.6%). Forty-four patients (62%) experienced recurrence. The mean time from the initial treatment to recurrence was 20 ± 33 months. The recurrence rate was 16/19 (84.2%) in patients who received medical therapy only, 11/17 (64.7%) in patients treated by surgery alone, and 17/31 (51.8%) in patients treated with surgery and medical therapy (*p* = 0.03). Conclusions: We observed a very high incidence of TES in Martinique. The factors associated with this high incidence in this specific geographical area remain to be elucidated. The frequency of recurrence was lower in patients who received both hormone therapy and surgery.

## 1. Introduction

Endometriosis is a disease of the female reproductive system that affects 5–10% of women of childbearing age [1]. It is a chronic disease, currently considered as a major public health problem in France. Endometriosis generally affects the pelvis, but the disease manifestations are diverse and varied [2] and include thoracic disease.

Extra-pelvic localization accounts for about 12% of endometriosis cases [3]. The most frequent extra-pelvic locations are abdominal and thoracic [4].

The pathophysiology of endometriosis is characterized by the implantation and growth of the endometrial gland and stroma outside the uterine cavity [5]. Endometriosis is now considered as a systemic disease, affecting various locations outside the female reproductive system [6]. Clinical symptoms include cyclic pelvic pain, deep dyspareunia and progressive dysmenorrhea, non-menstrual chronic pelvic pain, infertility, low body-mass index and mood disorders [6,7]. After clinical suspicion, ultrasound and/or magnetic resonance imaging (MRI) can help with assessment of endometriomas, fibroids, adenomyosis or other adnexal masses [6,8]. Definitive diagnosis is based on laparoscopy and histological confirmation [8]. The pathophysiology of endometriosis is based on theories and the pathogenesis remains poorly understood [9]. It is characterized by the ectopic localization of endometrial cells responsible for chronic inflammatory disease [6,10]. This is called a chronic inflammatory disease associated with immune dysfunction [6,11]. Several different pathogenetic pathways have been considered, including retrograde menstruation, benign metastasis, immune dysregulation, coelomic metaplasia, hormonal imbalance, involvement of stem cells and alterations in epigenetic regulation. It has also been suggested that there are interactions between the immune system, hormones, genes, local and stem cells [9]. Extragenital endometriosis can affect the gastrointestinal system (rectum, sigmoid colon, liver), urogenital system (bladder, ureters), respiratory system and nervous system [12,13]. Others locations may concern the umbilicus, breast and others [14].

Thoracic endometriosis syndrome (TES) is one of the possible life-threatening manifestations of endometriosis [15].

The manifestations of TES are catamenial pneumothorax, hemothorax, hemoptysis and pulmonary nodules, to which some studies add diaphragmatic hernia [16,17]. TES mainly occurs in the form of catamenial pneumothorax (CP), namely pneumothorax occurring during the perimenstrual period [4]. CP may be the first expression of pelvic endometriosis and TES can occurred in women without known pelvic endometriosis (PE) [18].

Extra-pelvic localization could be related to the retrograde migration of endometrial cells and their localization at the level of different organs. The chest is the most common extra-pelvic localization [4]. Thoracic manifestations involve the presence of endometrial tissue in the chest, and endometrial tissue has been reported to be found in the pulmonary parenchyma, pleural tissue, diaphragm and bronchi [15,16]. Three theories are proposed to explain the presence of intrathoracic endometrial implants [15]:

- Coelomic metaplasia;
- Lymphatic or hematogenous embolization from the uterus or pelvis;
- Retrograde menstruation with subsequent transperitoneal–transdiaphragmatic migration of endometrial tissue,

And four mechanisms are proposed to explain the pathogenesis of catamenial pneumothorax [15]:

- Spontaneous rupture of blebs;
- Transdiaphragmatic passage of air from the genital tract;
- Sloughing of endometrial implants from visceral pleura with subsequent air leak;
- Alveolar rupture caused by prostaglandin-induced bronchiolar constriction or by a check-valve mechanism exerted by bronchiolar endometrial implants.

In France, the incidence rate of spontaneous pneumothorax in subjects aged over 14 years is estimated at 22.7 cases per 100,000 inhabitants, with a female/male sex ratio of 1/3.3 [19]. In the United Kingdom, the incidence rate of spontaneous pneumothorax between 1991 and 1995 was estimated at 24 cases per 100,000 inhabitants for men, and 9.8 cases per 100,000 inhabitants for women.

Saito et al. reported that catamenial pneumothorax accounted for 18.6% of all female pneumothoraxes in Japan [20]. In France, Alifano et al. reported that catamenial pneumothorax rates accounted for 24.6% of pneumothoraxes in women [21].

Martinique is a department of the French West Indies, with an estimated population of 364,508 inhabitants in 2019, including 60,890 women aged from 15 to 45 years [22]. This population is dominated by Afro-descendant people. There is no data for the disease in Martinique. In our institution, which is the main referral hospital for Martinique, the occurrence of TES seems to be higher than in Metropolitan France. The environmental history of Martinique is marked by the use for several decades of chlordecone (sold as Kepone in the USA, or Merex in the UK or Curlone in France), a pesticide considered to be an endocrine disruptor, in banana plantations [23]. To date, some studies suggest a link between organochlorines (such as chlordecone) and endometriosis but this link is not confirmed [23,24].

To date, no data are available about the endometriosis or TES in the population of Martinique.

The main objective of this study was to describe the epidemiological and clinical characteristics, as well as the outcomes of patients with TES in Martinique.

## 2. Patients and Methods

We conducted an observational retrospective descriptive monocentric study including patients managed at Martinique University Hospital for TES from 1 January 2004 to 31 December 2020.

Inclusion criteria were as follows:

- Catamenial pneumothorax or endometriosis-related pneumothorax with per-operative findings or histological evidence;
- Catamenial hemothorax or endometriosis-related hemothorax with per-operative findings or histological evidence;
- Catamenial hemoptysis;
- Catamenial pulmonary nodule.

There were no other exclusion criteria except the absence of at least one of the inclusion criteria.

Patients were identified from the hospital informatics database using the following tenth revision of the International Classification of Diseases diagnostic codes: J93+N80, J91+N80, J92+N80, R04.2+N80, R91+N80, N80.8.

**Figure 1:**
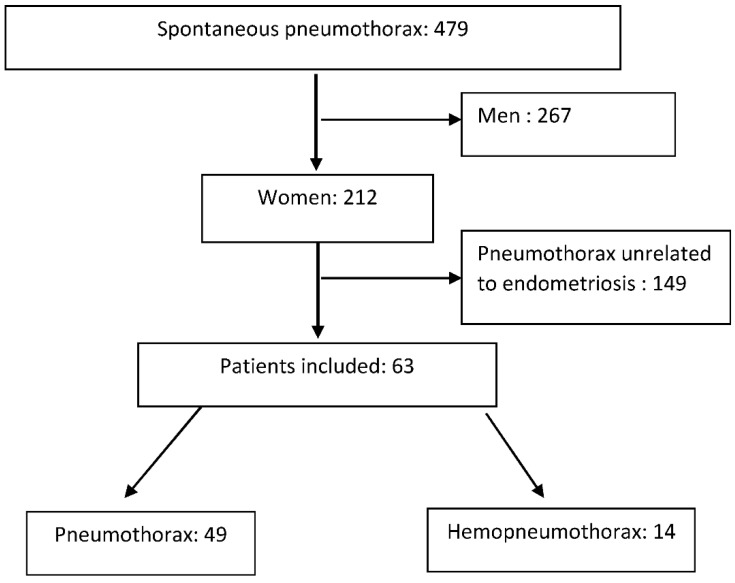
Flowchart of patient selection.

All admissions for spontaneous pneumothorax (SP) occurring in females were collected, and those associated with endometriosis were identified. We also collected endometriosis-related hemothorax and hemoptysis. Figure 1 shows the flowchart of patients with catamenial pneumothorax.

We recorded, for all the patients, baseline sociodemographic data, clinical data (history of pelvic endometriosis, age at diagnosis of pelvic endometriosis, number of pregnancies, number of births, symptom management), management strategies, and outcome after treatment. We recorded details of management, including the following strategies:

- Surgical management, via a thoracoscopic approach, consisting of exhaustive inspection, partial resection or prosthetic reinforcement of diaphragmatic lesion, resection of lung lesions if present, and systematic pleurodesis (mostly talc pleurodesis);
- Medical management, consisting of 6–12 months of prolonged hormone blockade, usually with Gonadotrophin Releasing Hormone (GnRH) analogues.

The first-line therapeutic strategy in cases of confirmed diagnosis in our center was to perform surgery with hormone blockade. Patients who could not benefit from medical treatment given an imminent pregnancy project, could benefit only from surgical treatment.

Recurrence was defined by the reappearance of a catamenial respiratory symptom with the presence of a compatible radiological abnormality without necessarily a need for specific management.

The primary endpoint was the incidence rate of TES in Martinique. Secondary endpoints were the prevalence rate of TES, incidence rate of CP and the rate of recurrence after treatment.

The study was performed in accordance with the Declaration of Helsinki and French legislation relating to research involving human beings. In French hospitals, patients are informed in writing that their data contained in their medical records can be used for retrospective research purposes. They have the right to refuse by notification either orally or in writing. If not, once these data are anonymized, the law allows their use with the approval of an ethics committee. This was the case in our study. Indeed, the patients’ data was rendered completely anonymous according to the requirements of the French National Authority for the Protection of Privacy and Personal Data (Commission nationale de l’informatique et des libertés, CNIL). The data was accessed and analyzed retrospectively from the University Hospital of Martinique. The Institutional Review Board of the University Hospitals of Martinique granted approval for the study (under the number 2020/062).

Descriptive analysis was performed. Quantitative variables were described as mean ± standard deviation (SD), and categorical variables as the number and percentage. Quantitative variables were compared using the Student’s *t*-test, and categorical variables using the chi square of Fisher’s exact test, as appropriate. Standardized incidence rates and prevalence rates were calculated using estimated population statistics from the French National Statistics Institute for 2019 (INSEE, Institut National de la Statistique et des Etudes Economiques). We considered the population of women of childbearing age to be those aged from 15 to 45 years.

Statistical analyses were performed using SAS version 9.4, (SAS Institute, Inc., Cary, NC, USA). Tests were considered as significant for *p*-values < 0.05.

### Patient and Public Involvement

Our study was retrospective and was carried out on the patient’s medical data. Patients included received an information letter and did not express any opposition to the use of their data, in accordance with French legislation on the matter. Patients were not involved in this study.

## 3. Results

### 3.1. Epidemiology

Over the study period, 864 patients were managed for endometriosis in our hospital. Over the study period, as described in Figure 1, 479 patients aged 15–45 years were admitted for spontaneous pneumothorax. Among these, 212 (44.3%) were females. Sixty-three female patients were included as having TES, including 14 hemopneumothorax and 49 pneumothorax. Eight patients had catamenial hemothorax. No case of isolated catamenial lung nodule or catamenial hemoptysis was found.

**Figure 2:**
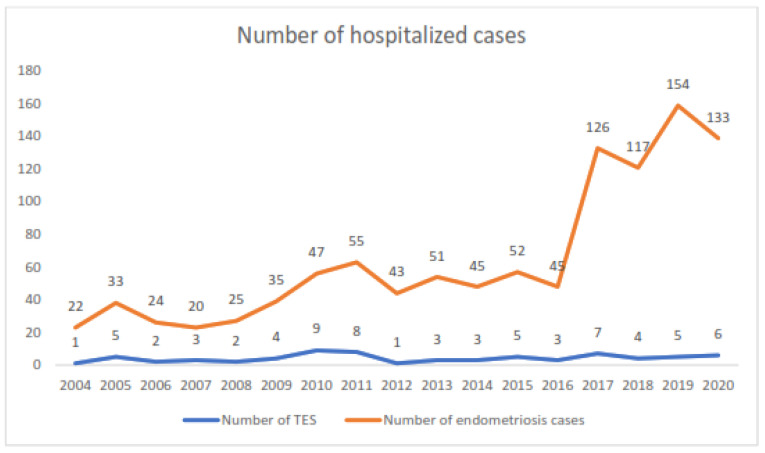
Annual number of cases hospitalized for TES and endometriosis over the study period.

The annual incidence rate of thoracic endometriosis (TES) was 1.1 patients/year/100,000 inhabitants. The annual number of cases is shown in Figure 2. We observed 63 patients with CP pneumothorax over a period of 17 years, for an estimated population of 364,508 inhabitants in 2019, yielding an annual person-based rate of 1 patient/100,000 inhabitants. With an estimated 60,890 women aged from 15 to 45 years in 2019, the annual incidence rate was estimated at 6.9/100,000 women in this age group, which corresponds to childbearing age.

Compared to the total population of Martinique in 2019 (estimated at 364,508 inhabitants), the standardized annual incidence rate of women’s spontaneous pneumothorax was 3.4 per 100,000 inhabitants.

### 3.2. Clinical Presentation

The main clinical characteristics of the study population are reported in Table 1. The mean age at diagnosis of TES was 36 ± 6 years. Eight patients had no history of pelvic endometriosis. The mean age at diagnosis of pelvic endometriosis was 29 ± 6 years. The median number of pregnancies and births was one (range 0–5) at the time of TES diagnosis. Only 19 patients reported having given birth, and 27 patients had had no pregnancy. The pregnancy status was missing for 25 patients.

### 3.3. Management

The average time from symptom onset to TES diagnosis was 24 ± 50 weeks, while the average time from TES diagnosis to surgery was 53 ± 123 days. Hemoptysis and pulmonary nodule in association with pneumothorax were each found in one case, but not proven histologically. Thirty-two patients (47%) had a history of abdominopelvic surgery. Seventeen patients (24%) presented other localizations (Table 2). Sixty-nine patients (97%) underwent surgery. Diaphragmatic nodules and/or perforations were found in 68/69 patients (98.5%). A total of 65 patients (91.5%) underwent diaphragmatic or pleural resection, and of these, histological confirmation was obtained in 55 (84.6%). Of note, during the first half of the study period, the pathology laboratory in our institution did not perform specific immunohistochemical analysis for the detection of endometrial cells.

### 3.4. Outcomes

Forty-four patients (62%) experienced recurrence. The mean time from the treatment to recurrence was 20 ± 33 months. The recurrence rate according to the clinical figure and management option is showed in Table 3: 16/19 (84.2%) in patients who received medical therapy only, 11/17 (64.7%) in patients treated by surgery alone and 17/31 (51.8%) in patients treated with surgery and medical therapy (*p* = 0.03).

Regarding the clinical presentation, 27/49 (55%) patients who initially presented with pneumothorax subsequently had recurrence; 16/22 (72.7%) with hemothorax or hemopneumothorax had recurrence (*p* = 0.3).

Recurrence was observed in the following circumstances:

- After discontinuation of medical treatment: seventeen cases (38.6%);
- Medical treatment without prior surgery: nine cases (20.5%);
- Surgery alone (without hormonal treatment): six cases (13.6%);
- Pleural evacuation (awaiting diagnostic confirmation): six cases (13.6%);
- Unknown: six cases (13.6%).

## 4. Discussion

In our population, the annual incidence of TES was 1.1 cases/year/100,000 inhabitants. The prevalence of TES was 1.2/1000 women aged from 15 to 45 years. CP concerned 30% of pneumothorax in women. The mean age of diagnosis was 29 years old for PE and 36 years old for TES. The main symptom was chest pain. The right side was concerned in 94% of cases. Recurrence occurred in 62% and was less in the patients treated with surgical and medical dual modality. 

TES is still considered as a rare clinical entity, but the relatively high frequency of observed cases is alarming, considering our population pool. As far as we know, no study to date has reported the incidence of TES on a large scale. Several consecutive series point out an increasing ratio of catamenial pneumothorax and thoracic endometriosis-related pneumothorax among spontaneous pneumothorax referred for surgery. In addition, three nationwide registries recently assessed the incidence and features of spontaneous pneumothorax. To compare our unusually high level of TES, we combined the observed data from the national cohorts with the endometriosis-related ratio reported by specialized teams, resulting in an extrapolated annual incidence for each territory (Table 4). According to Alifano et al. and their single-center consecutive experience over the past two decades, we focused on SP in women of childbearing age referred for surgery. Among these these childbearing women, Legras et al. [25] highlights a 41% correlation with TES. From a French registry of SP hospitalization over 4 years [19], we extracted 9773 women (18–50 years), in which 2699 (27.6%) underwent surgery. Applying the 41% ratio, we hypothesized that there would be 1106 cases of TES and deduced an annual hospitalization incidence of 0.42/100,000 population. The English registry [26] was published in 2018, but only the 2015 data was accessible, with 720 SP in women (15–49 years). We did not find any British study available concerning TES, and thus, we arbitrarily applied the French rates. In this sub-group, we estimated that 199 women would undergo surgery, 81 with TES and an annual person-rate incidence of 0.16/100,000 population. The nationwide Japanese study [27] recorded patients with SP over 4 years, from databases collecting 55% of hospitalizations in the country. They reported 11,599 women (13–53 years), of whom 4501 (38%) were referred for surgery, but only 813 with catamenial pneumothorax, and an annual person-base incidence of 0.22/100,000 population, despite their female specific peak around 40 years. Nevertheless, Ochi et al. [28] published a histopathological study collected from a single-center cohort in Tokyo. Among 435 women (15–55 years) operated on for SP, they diagnosed 160 (38%) with proven TES, which is similar to Alifano’s findings. Applying this rate to the Japanese registry, the annual person-base incidence would actually be 0.42/100,000 population (Table S1). We report quite a small cohort in Martinique, but due to our insularity, the strong limitations on air travel with SP, stable records during the study and with a single reference center for thoracic disease, we believe our data are representative of widespread TES on our island. The low sex ratio (M/W) of 1.25 in Martinique compared to 2.7–4.6 in other countries reinforces our findings about this high incidence of TES.

Regarding the age of diagnosis, Gil et al. found in a literature review that the diagnosis of catamenial pneumothorax was usually made at an older age than that of pelvic endometriosis [29], which is generally diagnosed between the ages of 24 and 29 on average, while TES is diagnosed about 5 years later [15,30]. Our results are therefore in line with those of the literature.

In their study, Gil et al. also observed right-sided pneumothorax in 93% of cases [29].

They also reported that 55% of patients had pelvic endometriosis, 50% with a prior pelvic intervention [29]. We found similar results in our study, where 47% had a history of prior abdominopelvic surgery, and 94% had right-sided pleural involvement. However, 83% of our patients had a history of pelvic endometriosis.

The management of thoracic endometriosis syndrome is multidisciplinary [1,16,31]. It combines hormonal and surgical treatment, if necessary [10]. Surgical treatment combines pleural symphysis and diaphragmatic repair. The disease course is often marked by recurrence, even in the case of surgical treatment [1,32]. We observed 62% recurrence, but the patients who underwent diaphragmatic repair and pleural talc often had minimal detachments, which did not require new surgery. Gil et al. reported 14.3–55% of recurrence of CP [29].

With regard to the medical history, 17.4% of our patients had no known history of pelvic endometriosis; this is not unusual, and indeed, higher rates have previously been reported elsewhere [18,29].

In terms of outcomes, we observed a recurrence rate of 62%, but after surgery, there was minimal recurrence not requiring new surgery. The main causes of recurrence were discontinuation of medical treatment, medical treatment alone or surgery alone. Dual therapy associating surgery with hormone blockade seemed to yield the lowest recurrence rates. Joseph et al. reported a recurrence rate that was higher after hormone therapy compared to surgery [30]. Fukuda et al. reported, as in our study, that the recurrence rate was lowest in the patients who received surgery followed by post-operative hormone therapy [33]. Elsewhere, Alifano et al. reported recurrent pneumothorax in 20 out of 35 women who presented with pneumothorax, of which 13 were idiopathic and 22 were related to endometriosis. They reported a recurrence rate of 17.1% after surgery followed by hormone therapy [34].

Martinique is a French overseas department in the French West Indies, and the majority of the population are Afro descendants. In a recent review, Bougie et al. found ethnic heterogeneity, with a diagnosis of endometriosis being less common in Blacks and Hispanics [35]. We currently have no data on the prevalence rate of endometriosis in Martinique and thoracic endometriosis in France, but our results suggest that the prevalence rate of thoracic endometriosis appears to be high in Martinique.

There are three possible explanations for this. First, there may be an increased regional incidence rate of endometriosis that is underdiagnosed. This hypothesis is supported by the high rate of early puberty and prematurity. Second, underdiagnosis results in the disease being undertreated, and TES is revealed at an advanced stage of the disease. The normal endometriosis prevalence rate that is underdiagnosed and undertreated in the early stages would induce more advanced-stage cases presenting as TES. Finally, women living in Martinique may be more susceptible to a chest localization, due to specific personal characteristics. The first hypothesis seems the most plausible.

Among the possible environmental factors hypotheses to be assessed is the use of chlordecone. Chlordecone is an endocrine disruptor that was used as a pesticide for several decades and contaminated the soil for several more decades in this region. The role of endocrine disruptors has been widely investigated, for example, with bisphenol A and other organochlorinated environmental pollutants [36,37]. However, the involvement of chlordecone in the epidemiology of endometriosis in our region is only a hypothesis and further investigation remains warranted, notably to determine the role of chlordecone and/or other demographic or environmental factors in the occurrence of endometriosis.

### Strengths and Limitations

Our study is the first epidemiological study to be performed in this region. It is a population-based study that gives insights into the epidemiology and frequency of TES in Martinique. Even though this study did not investigate the epidemiology of pelvic endometriosis or other localizations (which also deserve to be studied), it had the advantage of having specifically explored pleural and pulmonary disorders.

The limitations include the retrospective nature of the study, which precludes controlling for potential confounders. There was some missing data, notably regarding pregnancies, deliveries and the time from symptom onset to diagnosis. Finally, our study involved a population with a majority of women of African descent, and the findings may therefore not be generalizable to populations of other ethnic origins.

## 5. Conclusions

We report an extremely high incidence rate of thoracic endometriosis syndrome, suggesting a high prevalence rate of the disease in Martinique. Pneumothorax was the most common manifestation. CP accounts for a third of pneumothorax for women. Recurrence was frequent for TES patients especially when they stopped hormonal therapy. Dual therapy combining surgery and hormone blockade yielded the lowest recurrence rates. Investigation of environmental or other factors remains necessary to advance our understanding of the epidemiology of this disease in our region.

## Figures and Tables

**Table 1:** Clinical and demographic characteristics and outcome of patients managed for thoracic endometriosis syndrome.

Characteristics	Mean	Standard Deviation	Missing Data
Age at diagnosis of TES	36	6	1
Age at diagnosis of pelvic endometriosis	29	6	9
Number of pregnancies	1	1	28
Number of births	1	1	25
Time from symptom onset to diagnosis (weeks)	24	50	17
Time to recurrence (months)	20	33	0
Medical history			
	*n*	%	
Tobacco/cannabis use	6	12.0	7
History of pelvic endometriosis	57	82.6	2
Previous contraception	25	38.5	6
History of abdominopelvic surgery	32	47.1	3
Other localization	17	24.0	0
Main presenting symptom			
Dyspnea	32	45.1	0
Chest pain	38	53.5	
Cough	1	1.4	
Sidedness			
Right	67	94.4	0
Left	4	5.6	0
Initial treatment			
Medical treatment	20	28.2	0
Surgical treatment	18	25.4	0
Combined treatment	33	46.5	0

**Table 2:** Other extra-pelvic and extra-thoracic localizations of endometriosis.

Localizations *n* = 17	*n*	%
Peritoneum	8	47.1
Umbilical	3	17.6
Colon	2	11.8
Disseminated	2	11.8
Bladder	1	5.9
Liver	1	5.9

**Table 3:** Recurrence according to clinical presentation and treatment option.

Recurrence	*n*	%	*p*
Medical treatment *n* = 19	16	84.2	0.03
Surgery *n* = 17	11	64.7	
Combined *n* = 31	17	51.8	
Pneumothorax *n* = 49	27	55.0	0.3
Hemothorax/hemopneumothorax *n* = 22	16	72.2	

**Table 4:** Comparative incidence of SP and TES.

	SP Annual Incidence/100,000	Women SP Annual Incidence/100,000	Sex Ratio (M/W)	TES Annual Incidence/100,000
France	22.7	5.3	3.3/1	0.42 *
England	14.0	7.6	2.7/1	0.16 *
Japan	37.2	6.6	4.7/1	0.42 *
Martinique	7.7	3.4	1.3/1	1.14

M: men, W: women, * Estimated incidence.

## Data Availability

The datasets generated and/or analyzed during the current study are available from the corresponding author on reasonable request.

## Supplementary Materials

The following supporting information can be downloaded at: https://www.mdpi.com/article/10.3390/jcm12175578/s1, Table S1: Estimated annual incidence of SP and TES in France, England and Japan. References [19,25,26,27,28] are cited in Supplementary Materials.

## Abbreviations

CHU: University Hospital. CP: Catamenial pneumothorax. GnRH: Gonadotropin-Releasing Hormone. PE: Pelvic endometriosis. TES: Thoracic endometriosis syndrome.
